# Is atelectasis related to the development of postoperative pneumonia? a retrospective single center study

**DOI:** 10.1186/s12871-023-02020-4

**Published:** 2023-03-11

**Authors:** Eunji Ko, Kyung Yeon Yoo, Choon Hak Lim, Seungwoo Jun, Kaehong Lee, Yun Hee Kim

**Affiliations:** 1 Department of Anesthesiology and Pain Medicine, Korea University Anam Hospital, 73, Goryeodae-ro, Seongbuk-gu, Seoul, 02841 Republic of Korea; 2 Department of Anesthesiology and Pain Medicine, Chonnam National University Hospital, 42 , Jebong-ro, Dong-gu, Gwangju, 58128 Republic of Korea; 3 Department of Anesthesiology and Pain Medicine, College of Medicine, Korea University, 73, Goryeodae-ro, Seongbuk-gu, Seoul, 02841 Republic of Korea; 4 Department of Anesthesiology and Pain Medicine, Hanyang University Changwon Hanmaeum Hospital, 57, Yongdong-Ro, Uichang-Gu, Gyeongsangnam-Do, Changwon-Si, 51139 Republic of Korea

**Keywords:** General anesthesia, Postoperative pneumonia, Postoperative pulmonary complications, Pulmonary atelectasis, Surgery

## Abstract

**Background:**

Atelectasis may play a substantial role in the development of pneumonia. However, pneumonia has never been evaluated as an outcome of atelectasis in surgical patients. We aimed to determine whether atelectasis is related to an increased risk of postoperative pneumonia, intensive care unit (ICU) admission and hospital length of stay (LOS).

**Methods:**

The electronic medical records of adult patients who underwent elective non-cardiothoracic surgery under general anesthesia between October 2019 and August 2020 were reviewed. They were divided into two groups: one who developed postoperative atelectasis (atelectasis group) and the other who did not (non-atelectasis group). The primary outcome was the incidence of pneumonia within 30 days after the surgery. The secondary outcomes were ICU admission rate and postoperative LOS.

**Results:**

Patients in the atelectasis group were more likely to have risk factors for postoperative pneumonia including age, body mass index, a history of hypertension or diabetes mellitus and duration of surgery, compared with those in the non-atelectasis. Among 1,941 patients, 63 (3.2%) developed postoperative pneumonia; 5.1% in the atelectasis group and 2.8% in the non-atelectasis (*P* = 0.025). In multivariable analysis, atelectasis was associated with an increased risk of pneumonia (adjusted odds ratio, 2.33; 95% CI: 1.24 – 4.38; *P* = 0.008). Median postoperative LOS was significantly longer in the atelectasis group (7 [interquartile range: 5–10 days]) than in the non-atelectasis (6 [3–8] days) (*P* < 0.001). Adjusted median duration was also 2.19 days longer in the atelectasis group (β, 2.19; 95% CI: 0.821 – 2.834; *P* < 0.001). ICU admission rate was higher in the atelectasis group (12.1% vs. 6.5%; *P* < 0.001), but it did not differ between the groups after adjustment for confounders (adjusted odds ratio, 1.52; 95% CI: 0.88 – 2.62; *P* = 0.134).

**Conclusion:**

Among patients undergoing elective non-cardiothoracic surgery, patients with postoperative atelectasis were associated with a 2.33-fold higher incidence of pneumonia and a longer LOS than those without atelectasis. This finding alerts the need for careful management of perioperative atelectasis to prevent or reduce the adverse events including pneumonia and the burden of hospitalizations.

**Trial registration:**

None.

## Introduction

Surgery and general anesthesia are likely to impair the respiratory function, thus increasing the incidence of postoperative pulmonary complications (PPCs) [1]. PPCs, particularly atelectasis and pneumonia, are major causes of postoperative morbidity and mortality [2–4], along with prolonged hospital length of stay (LOS) and increased resource utilization and healthcare expenditure [1]. Atelectasis may occur shortly after preoxygenation and induction of general anesthesia, leading to an intrapulmonary shunt of blood through non-ventilated lung tissue and ventilation/perfusion mismatch. It may in turn compromise gas exchange in most patients (90%) undergoing surgery under general anesthesia. Although atelectasis generally affects a small portion of dependent lung regions, it may be more pronounced in patients who are obese, undergoing thoracic or open abdominal surgery, or in the Trendelenburg or lateral decubitus position [5, 6]. It may then contribute to the development of infectious complications including pneumonia [7].

In an experimental study, amelioration of atelectasis with exogenous surfactants or open lung ventilation attenuated growth and translocation of bacteria administered into the trachea, and diminished the risk of pneumonia in a piglet model [8]. These findings are in line with those in a previous study, in which mechanical ventilation with zero positive end-expiratory pressure (PEEP) aggravated lung bacterial burden and deteriorated the histological aspects of pneumonia following the intrabronchial bacterial instillation, along with higher degree of atelectasis, when compared with spontaneously breathing controls, in rabbits [9]. However, the clinical evidence in human patients has been contradictory.

Protective intraoperative mechanical ventilation (low tidal volumes with appropriate levels of PEEP) as compared with conventional ventilation (high tidal volumes with no PEEP) reduced the occurrence of PPCs. In patients undergoing major abdominal surgery, low tidal volumes and low levels of PEEP with alveolar recruitment maneuvers also reduced the risk of major pulmonary complications and health care utilization [4]. On the contrary, when the low tidal volumes were used during surgery, a higher level of PEEP with alveolar recruitment maneuvers did not reduce the incidence of PPCs [10–12], nullifying the protective ventilatory strategy. In fact, previous studies on PPCs have never analyzed postoperative pneumonia as a primary outcome of atelectasis, and only collectively evaluated atelectasis and pneumonia as a composite of PPCs.

Admission of patients to the intensive care unit (ICU) who are at high risk of subsequently requiring physiological support is considered a standard of care in many healthcare systems. However, ICU beds are costly and of limited resource [13]. In addition, the length of time spent hospitalized well represents the degree of hospital resources utilized, such as bed occupancy, staffing, and equipment [14]. To date, no studies have evaluated the relationship between postoperative atelectasis and patient outcomes including ICU admission and LOS. The present study was aimed to determine whether the presence of atelectasis is related to an increased incidence of postoperative pneumonia, ICU admissions, and LOS in patients who underwent elective non-cardiothoracic surgery under general anesthesia. The primary outcome was the development of pneumonia. The secondary outcomes were ICU admission, and postoperative LOS.

## Methods

### Study design and selection of participants

This retrospective study was approved by the Institutional Review Board of Korea University Anam Hospital (IRB No. 2020AN0507), which waived the need for informed consent, because it was retrospective and without any risk to patients. Electronic medical records were reviewed to determine the incidence of postoperative pneumonia, ICU admission rate and postoperative LOS in patients with atelectasis. All methods were carried out in accordance with current regulations and guidelines.

Patients aged 18 years or older who underwent elective non-cardiothoracic surgery under general anesthesia at Korea University Anam Hospital between October 1, 2019, and August 31, 2020, were screened. Among them, patients who had chest radiographs (CXRs) taken within the first seven postoperative days were included. Patients were then excluded from the analysis if they 1) had preoperative fever or uncertainty about infection of other organs, 2) had atelectasis on CXR taken preoperatively, 3) had preoperative neuromuscular disease, 4) had chest tubes, endotracheal tube or tracheostomy before or after the surgery, and 5) had two or more operations within 4 weeks after surgery. We did not include patients who underwent cardiothoracic surgery because of the higher incidence of postoperative pneumonia compared to non-cardiothoracic surgery [15] in this study.

### Variables

Demographic and clinical variables obtained included age, sex, body mass index (BMI), comorbidities (asthma, chronic obstructive lung disease [COPD], hypertension, heart failure, diabetes mellitus, and anemia), current smoking status, selected laboratory results (i.e. albumin), American Society of Anesthesiologists (ASA) physical status, pre- and postoperative CXRs, type of surgery, duration of anesthesia, type of reversal agent for neuromuscular blockade, postoperative occurrence of atelectasis and pneumonia, ICU admission, and postoperative LOS. The subjects were divided into two groups, one with postoperative atelectasis (atelectasis group) and the other without (non-atelectasis group), to explore the effects of atelectasis on the outcomes.

### Outcomes

The incidence of pneumonia that occurred within the first 30 days following surgery was evaluated as the primary outcome, as recommended as a follow-up period for the occurrence of adverse events in perioperative medicine [16]. The secondary outcomes were ICU admission rate and postoperative LOS. Atelectasis was defined as lung opacification with a shift of the mediastinum, hilum, or hemidiaphragm toward the affected area, and compensatory overinflation in the adjacent non-atelectatic lung on CXR obtained within the first 7 postoperative days. Radiological diagnoses were reported by the attending radiologists independent of the study. Preoperative CXR is a routine evaluation in every department while postoperative CXR is routinely obtained in a few departments or as required when a patient have fever, or develops respiratory symptoms, including dyspnea, shortness of breath, cough and sputum, or decrease of oxygen saturation in our hospital.

Postoperative pneumonia was diagnosed by respiratory physicians as consulted by the clinicians in charge of the patients based on the US Centers for Disease Control definition of pneumonia with two or more serial CXR showing at least one of the following findings: (i) new or progressive and persistent infiltrates, (ii) consolidation, and (iii) cavitation (one radiograph is sufficient for patients with no underlying pulmonary or cardiac disease). They also should have at least one of the following signs: (i) fever (> 38℃) with no other recognized cause; (ii) leukopenia (white cell count < 4 × 10^9^ /L) or leukocytosis (white cell count > 12 × 10^9^ /L), (iii) altered mental status with no other recognized cause for adults > 70 years old; and at least two of the following: (i) new onset of purulent sputum or change in character of sputum, or increased respiratory secretions, or increased suctioning requirements, (ii) new onset or worsening cough, dyspnea, or tachypnea, (iii) rales or bronchial breath sounds, and (iv) worsening gas exchange (hypoxemia, increased oxygen requirement, increased ventilator demand) [16, 17].

### Anesthetic management

Patients are premedicated with midazolam (0.1 mg/kg, p.o.) 60 min before induction of anesthesia. After full preoxygenation, anesthesia is induced with propofol and remifentanil in the inhalation anesthesia as well as in the total intravenous anesthesia. After administration of rocuronium 0.6 – 1.0 mg/kg i.v., the trachea is intubated and the lungs are mechanically ventilated with air and oxygen at 0.5 fraction of inspired oxygen with or without the use of PEEP (5 cmH_2_O) in all subjects. Anesthesia is maintained using sevoflurane or desflurane combined with remifentanil in the inhalation anesthesia and using propofol and remifentanil in the total intravenous anesthesia. Upon completion of the surgery, the anesthetic is discontinued, and residual neuromuscular block is antagonized with pyridostigmine and glycopyrrolate or sugammadex. Postoperative interventions such as chest physiotherapy, bronchodilators and antibiotics, which may have an impact on the development of atelectasis and pneumonia are not provided as standard of care for surgical patients in our hospital.

### Sample size and statistical analysis

The incidence of postoperative pneumonia known to date is as low as 1 ~ 2% [18, 19] and as high as 28.9% [20]. In a pilot study of patients who underwent non-cardiothoracic surgery during 5 days in the first week of April 2020, 11 cases (20.0%) of atelectasis and 2 cases (3.6%) of pneumonia were observed in 55 patients, among 174 patients screened. Assuming that 3.6% of the 55 patients would develop postoperative pneumonia, at least a sample of 160 patients was required to detect a correlation between two independent variables with different incidence rates (20.0% vs. 3.6%), with a type 1 error of 0.05 and a power of 90% [21]. Since regression analysis was performed for 16 types of covariates affecting the incidence of pneumonia, we calculated a sample size of 2,560 subjects. In the pilot study, 55 out of 174 subjects screened satisfied the inclusion criteria; therefore, we planned to collect data from approximately 8,000 patients.

Data are expressed as total numbers (percentage) for categorical variables and as mean ± standard deviation or median (interquartile range [IQR]) as appropriate for continuous variables. Continuous variables were compared using univariate logistic regression, and categorical variables were compared using the chi-squared test or Fisher’s exact test as appropriate. Initially, covariates associated with the response variables (incidence of pneumonia and ICU admission rate) were screened. Covariates with a *P*-value < 0.2 as determined by univariate regression were included in the multivariate logistic regression analysis using stepwise backward selection on a criterion of *P*-value < 0.05. Binary regression analysis was performed for pneumonia and ICU admission, where the dependent variables were dichotomous, and linear multiple regression analysis was performed for postoperative LOS, where the dependent variable was continuous. Statistical significance was defined as *P*-value < 0.05. Data were analyzed using IBM SPSS Statistics version 25 (IBM, Chicago, IL, USA).

## Results

**Fig. 1:**
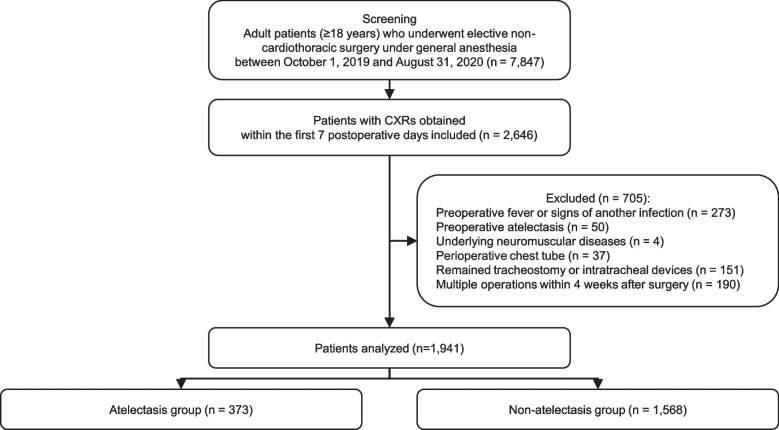
Patient screening and exclusion process

Patients who underwent elective non-cardiothoracic surgery under general anesthesia (aged ≥ 18 years) were screened, and 2,646 out of 7,847 patients met the eligibility criteria. Among them, 705 patients were then excluded because 273 had preoperative fever or signs of infection, 50 had preoperative atelectasis, 4 had neuromuscular diseases, 37 had chest tube before or after surgery, 151 had tracheostomy or intratracheal tube before or after surgery, and 190 underwent multiple surgeries within 4 weeks after the previous surgery. Therefore, a total of 1,941 patients were finally analyzed; 373 and 1,568 in the atelectasis and the non-atelectasis groups, respectively (Fig. 1).

The demographic and clinical characteristics of the enrolled patients are summarized in Table 1. Patients aged 60.2 [IQR, 51.0—72.0] years with 40.1% of women and high comorbidities. The atelectasis group aged more (63.3 ± 13.3 vs. 59.5 ± 16.3 years, *P* < 0.001), had higher BMI (25.6 ± 4.3 vs. 24.4 ± 4.4 kg/m^2^, *P* < 0.001], and was associated with more frequent preoperative diagnoses of hypertension (54.2 vs. 43.8%, *P* < 0.001) and diabetes mellitus (29.5 vs. 21.2%, *P* = 0.001) than the non-atelectasis. The atelectasis group was more likely to undergo upper abdominal surgery (*P* < 0.001) and longer surgical procedures (234.3 ± 113.2 vs. 186.6 ± 102.0 min; *P* < 0.001) and more frequently used sugammadex (70.5 vs. 51.5%, *P* < 0.001). Of 1,941 patients, postoperative CXRs were obtained routinely in 58.4%, due to postoperative fever in 29.3%, due to respiratory symptoms in 8.7%, and etc. (e.g. pre/post hemodialysis, upper gastrointestinal series) in 3.6%.

**Table 1:** Demographics and clinical characteristics of patients with or without atelectasis

Characteristics	Overall *n* = 1,941	Atelectasis group *n* = 373	Non-atelectasis group *n* = 1,568	*P*-value
Demographics				
Age, yr	60.2 ± 15.8	63.3 ± 13.3	59.5 ± 16.3	< 0.001
Sex (% male)	778 (40.1)	158 (42.4)	620 (39.5)	0.318
BMI, kg/m^2^	24.6 ± 4.4	25.6 ± 4.3	24.4 ± 4.4	< 0.001
Medical comorbidities				
Asthma	54 (2.8)	9 (2.4)	45 (2.9)	0.630
COPD	32 (1.6)	7 (1.9)	25 (1.6)	0.700
Hypertension	888 (45.7)	202 (54.2)	686 (43.8)	< 0.001
Heart failure	72 (3.7)	16 (4.3)	56 (3.6)	0.510
Diabetes mellitus	442 (22.8)	110 (29.5)	332 (21.2)	0.001
Anemia (hemoglobin < 10 g/dL)	168 (8.7)	36 (9.7)	132 (8.4)	0.447
Hypoalbuminemia (serum albumin < 3.5 g/dL)	182 (9.4)	34 (9.1)	148 (9.4)	0.847
Smoking history	340 (17.5)	77 (20.6)	263 (16.8)	0.077
Preoperative CXR abnormality	189 (9.7)	35 (9.4)	154 (9.8)	0.798
ASA physical status				0.070
I	83 (4.3)	6 (1.6)	77 (4.9)	
II	1364 (70.3)	265 (71.0)	1099 (70.1)	
III	450 (23.2)	93 (24.9)	357 (22.8)	
IV	44 (2.3)	9 (2.4)	35 (2.2)	
Type of surgery				< 0.001
Brain surgery	55 (2.8)	12 (3.2)	43 (2.7)	
Back and spine surgery	308 (15.9)	44 (11.8)	264 (16.8)	
Upper abdominal surgery	330 (17.0)	106 (28.4)	224 (14.3)	
Lower abdominal surgery	744 (38.3)	170 (45.6)	574 (36.6)	
Orthopedic surgery	416 (21.4)	33 (8.8)	383 (24.4)	
Other surgeries	88 (4.5)	8 (2.1)	80 (5.1)	
Anesthesia time, min	195.8 ± 105.9	234.3 ± 113.2	186.6 ± 102.0	< 0.001
Type of reversal agents				< 0.001
Pyridostigmine	848 (43.7)	108 (29.0)	740 (47.2)	
Sugammadex	1071 (55.2)	263 (70.5)	808 (51.5)	
None	22 (1.1)	2 (0.5)	20 (1.3)	
Postoperative pneumonia	63 (3.2)	19 (5.1)	44 (2.8)	0.025
ICU admission	147 (7.6)	45 (12.1)	102 (6.5)	< 0.001
Postoperative LOS, days	6 [4-8]	7 [5-10]	6 [3-8]	< 0.001
30-day mortality	5 (0.3)	0 (0.0)	5 (0.3)	0.275

Values are presented as mean ± standard deviation or median [interquartile range], for continuous variables and as total numbers (percentage) for categorical variables. Continuous variables were compared using univariate logistic regression, and categorical variables were compared using the chi-square test or Fisher’s exact test, as appropriate

*BMI* Body mass index, *COPD* Chronic obstructive pulmonary disease, *CXR* Chest radiography, *ASA* American society of anesthesiologists, *LOS* Length of hospital stay, *ICU* Intensive care unit

Pneumonia occurred in 63 cases (3.2%) within the first 30 days after the surgery: 19 of 373 patients in the atelectasis group (5.1%) and 44 of 1,568 patients in the non-atelectasis (2.8%) (*P* = 0.025). Multivariable regression analysis revealed that atelectasis (atelectasis vs. non-atelectasis) was an independent predictor for pneumonia (adjusted odds ratio, 2.33; 95% confidence interval (CI): 1.24 – 4.38; *P* = 0.008) (Table 2). Among the 1,941 patients, 145 (7.5%) were admitted to ICU after the surgery, with a rate significantly higher in the atelectasis group than in the non-atelectasis (12.1 vs. 6.5%, *P* < 0.001). However, after adjustment for confounders, no increase in ICU admission risk was observed (adjusted odds ratio, 1.68; 95% CI: 0.99 – 2.86; *P* = 0.054). The duration of postoperative LOS was significantly longer in the atelectasis group than in the non-atelectasis (median, 7 [IQR, 5–10] days vs 6 [3–8] days; *P* < 0.001). Adjusted median duration was 2.19 days longer in the atelectasis group (unstandardized regression coefficient, 2.19; 95% CI: 0.821 – 2.834; *P* < 0.001). Five patients died within 30 days after the surgery: 0 in in the atelectasis group and 5 in the non-atelectasis group (*P* = 0.275).

**Table 2:** Potential predictors of postoperative pneumonia among patients undergoing elective non-cardiothoracic surgery

Variables	Odds ratio	95% confidence interval	*P*-value
BMI, kg/m^2^	0.887	0.823 – 0.956	0.002
Asthma	3.032	1.020 – 9.011	0.046
Hypertension	2.583	1.342 – 4.971	0.004
Hypoalbuminemia	4.270	2.319 – 7.861	0.000
ASA physical status (as continuous variable)	3.050	1.978 – 4.704	0.000
Type of surgery, relative to lower abdominal surgery			
Brain surgery	3.614	0.988 – 13.225	0.052
Back and spine surgery	3.400	1.324 – 8.729	0.011
Upper abdominal surgery	1.872	0.781 – 4.488	0.160
Orthopedic surgery	2.277	0.962 – 5.390	0.061
Other surgeries	4.145	1.350 – 12.723	0.013
Type of reversal agents, relative to pyridostigmine			0.071
Sugammadex	1.645	0.861 – 3.143	0.132
None	5.454	1.119 – 26.587	0.036
Postoperative atelectasis	2.334	1.244 – 4.377	0.008

Results are from fully adjusted multivariate analysis with backward elimination (Wald test) accounting for all baseline characteristics

*BMI* Body mass index, *ASA* American society of anesthesiologists

## Discussion

The present study demonstrated that patients with postoperative atelectasis had a 2.33-fold higher risk of postoperative pneumonia compared with those without. The atelectasis was also associated with an extended LOS. However, it was not related to ICU admission rate after adjustment for confounders.

We found that patients with atelectasis were at increased risk of postoperative pneumonia but were also predisposed by independent risk factors to postoperative pneumonia including age [3, 19, 22], BMI [3, 23], a history of hypertension [23] or diabetes mellitus [23] and duration of surgery [22, 24] in our study. Nevertheless, adjustment for these risk factors still revealed direct association between the postoperative atelectasis and the risk of postoperative pneumonia. Our findings are important as the postoperative pneumonia might be preventable, at least in part, by reducing or reversing atelectasis in the perioperative period. In fact, intraoperative atelectasis during general anesthesia is known to be prevented by limiting the fraction of inspired oxygen (“absorption atelectasis”) [25] and by promoting alveolar recruitment with PEEP and recruitment maneuvers [26]. In addition, postoperative atelectasis has been reduced by using individual PEEP settings [27], and by maintaining a consistent positive pressure at the airway with pressure support and PEEP during emergence from general anesthesia as well [28]. To the best of our knowledge, this is the first report to strongly suggest postoperative atelectasis as a risk factor for pneumonia in surgical patients.

It has been known that atelectasis may induce local immune dysfunction and inflammation, thereby increasing the susceptibility to infection. In addition, perioperative changes in lung mechanics and breathing patterns due to general anesthesia and surgery and/or consequent atelectasis are known to weaken the lung defense system [29, 30], compromising both cough and mucociliary clearance against pathogens distal to the obstruction [31, 32]. Local depletion or dysfunction of surfactant due to a significant atelectasis, the use of anesthetic agents [33], and/or prolonged mechanical ventilation [34] also compromise the protective response against infection because surfactant has direct antimicrobial properties [35]. Taken together, compromised local immune system, impaired mucociliary clearance, and surfactant dysfunction appears to be responsible for some of the increased risk of pneumonia in post-surgical patients with atelectasis. Furthermore, atelectasis was shown to reduce the penetration of antibiotics into the affected lung, making it difficult to obtain the correct drug concentrations to fight against potential pathogens [36].

A recent meta-analysis demonstrated that a high inspired oxygen fraction (versus a low inspired oxygen fraction) had no effect on the incidence of pneumonia despite the increased incidence of postoperative atelectasis in patients undergoing non-thoracic surgery under general anesthesia [37]. Three possible mechanisms have been proposed to explain the atelectasis: resorption of alveolar air (i.e. absorption atelectasis), direct compression of lung tissue (i.e. compression atelectasis), and surfactant impairment [38]. The atelectasis was mainly attributed to absorption of alveolar air that spontaneously resolves within 24 to 48 h in a previous study [37]. On the other hand, upper abdominal surgery, which favors the development of compression atelectasis, was more frequent and the duration of general anesthesia eliciting both forms of atelectasis (i.e. surfactant dysfunction and compression) [38] was longer in the atelectasis group than in the non-atelectasis in our study. The discrepancy between the studies may be accounted for by different pathogenic mechanisms underlying the development of atelectasis.

The reported incidence of postoperative pneumonia varies widely by the site and type of surgery performed from 0.5% to 28.9% [18, 20, 39]. The incidence in our study (3.2%) was higher than that (1.8%) in ASA status III patients undergoing non-cardiothoracic predominantly abdominal and pelvic surgery [39]. The subjects were limited to 7-day postoperative follow-up in their study, whereas ours were to 30-day. In addition, 38.0% of postoperative CXRs were obtained from patients with symptoms of respiratory diseases including fever, shortness of breath, and cough. It has been reported that atelectasis itself does not complicate pneumonia, but may contribute to the development of pneumonia when exposed to infectious agents [32]. Since the fever that accompanies atelectasis is related to infection distal to the obstructed airway [40, 41], atelectasis may have progressed to pneumonia in some patients with postoperative CXR. Alternatively, it is also possible that pneumonia might have led to atelectasis because the presence of fever may reflect infectious complications rather than atelectasis. A longer follow-up period and bias in patient selection may explain why the incidence of pneumonia in our study was higher than that in a previous one [39].

Reversal of neuromuscular blockade with sugammadex was associated with a lower incidence of major PPCs, including atelectasis and pneumonia compared to the use of anticholinesterase in patients undergoing non-cardiac surgery in a previous study [42]. In contrast, the use of sugammadex resulted in a higher incidence of atelectasis compared to the use of anticholinesterase in our study (25.9% vs. 13.9%, *P* < 0.001), although it had no significant effect on pneumonia (3.7% vs. 2.6%, *P* = 0.201). In our hospital, sugammadex is generally used for patients who are at high risk for PPCs (e.g. upper abdominal or thoracic surgery), particularly in the elderly, whereas reversal agent options are currently limited by price. The bias in treatment and patient selection may explain the discrepancy between the studies. In fact, upper abdominal surgeries were more frequent [19] and the patients were older [24] in the atelectasis group compared to the non-atelectasis in our study, both of which are indeed risk factors for atelectasis.

Although the atelectasis seen during anesthesia resolves within 24 h after laparoscopy in non-obese subjects [43], it may persist for at least 24 h in most morbidly-obese patients [43] or with major surgeries [44]. A meta-analysis of postoperative atelectasis in a heterogeneous group of patients demonstrated CXR evidence of atelectasis in 57% of patients, with little improvement on the third day following the surgery [45]. By contrast, the incidence of atelectasis determined by CXRs obtained at various time points from the first to the 7th postoperative day was 19.2% (373/1,941), being much lower in our study. Moreover, 38.0% of postoperative CXRs were obtained because of respiratory symptoms including fever, which could lead to bias in patient selection in our study. Large prospective studies in which chest images are obtained early after surgery in every patient are needed to precisely determine the role of atelectasis in the development of pneumonia.

Our study has several limitations. First, it is a single center study, and there may be a lack of representativeness of the entire population. Second, being a retrospective study, it is not free from bias in patient selection and treatment. Not all covariates were controlled, nor were demographic and clinical characteristics. Third, the intraoperative use of high oxygen concentration or no PEEP is known to be associated with increased severity of atelectasis [46]. However, anesthetic management during the surgery was not harmonized among patients, and every patient followed routine anesthetic management in our hospital. Fourth, CXR, an inexpensive and the most frequently ordered radiological test, was used to determine atelectasis, resulting in some under-estimation of actual atelectasis cases. Chest CT scans have shown greater diagnostic sensitivity in patients undergoing lower abdominal surgery compared with CXR [44] and remains the accepted standard when measuring atelectasis. In addition, lung ultrasound was shown to be superior to CXR in diagnosing PPCs following cardiothoracic surgery. It has been widely used since it is portable, dynamic, and free of radiation [28, 47, 48]. Finally, we did not control for baseline lung function and did not report on the specific interventions used to manage postoperative atelectasis, which limits the ability to make specific recommendations for clinical practice.

In conclusion, our retrospective study demonstrated that patients with postoperative atelectasis had a 2.33-fold higher risk of postoperative pneumonia than those without. Moreover, the development of postoperative atelectasis was associated with a prolonged LOS, while not being with ICU admission. Clinicians should try to prevent or treat postoperative atelectasis to reduce devastating adverse events including postoperative pneumonia, and to decrease resource utilization and healthcare expenditure in patients undergoing elective non-cardiothoracic surgery. Atelectasis as a risk factor for pneumonia warrants further investigation.

## Data Availability

The data generated and analyzed during the current study are not publicly available, but are available from the corresponding author on reasonable request.

## Abbreviations

*ASA*: American Society of Anesthesiologists
*COPD*: Chronic obstructive pulmonary disease
*CT*: Computed tomography
*CXR*: Chest radiography
*EMR*: Electronic medical record
*ICU*: Intensive care unit
*LOS*: Length of hospital stay
*OR*: Odds ratio
*PPC*: Postoperative pulmonary complication
*SD*: Standard deviation
